# Effects of electroacupuncture on pediatric chronic urinary retention: a case-series study

**DOI:** 10.3389/fped.2023.1194651

**Published:** 2023-07-21

**Authors:** Min Yang, Shuai Gao, Hao Yao, Xin He, Jiufei Fang, Yu Chen, Zhishun Liu

**Affiliations:** ^1^ Department of Acupuncture, Guang'anmen Hospital, China Academy of Chinese Medical Sciences, Beijing, China; ^2^ Graduate School, Beijing University of Chinese Medicine, Beijing, China; ^3^ Beijing Houpo Chinese Medicine Institute, Beijing, China

**Keywords:** chronic urinary retention, lumbosacral surgeries, electroacupuncture, pediatric patients, case-series

## Abstract

**Objectives:**

This study aims to preliminarily evaluate the effect and safety of electroacupuncture (EA) in treating pediatric chronic urinary retention (CUR) following lumbosacral surgeries, with treatment duration evaluated.

**Methods:**

This prospective case-series study was performed from August 5, 2017, to July 31, 2022. Pediatric patients diagnosed with CUR following lumbosacral surgeries were included and treated by EA for 2–16 weeks. Responders were defined as participants achieving a reduction of 50% or more in post void residuals (PVR) from baseline. Time-to-event analysis was applied to explore the association between EA treatment duration and response rate. Adverse event was recorded.

**Results:**

Totally 14 participants (mean [SD] age, 12 [4] years) completed EA treatment. Response rate was 71% (10/14) at the 12th week. 50% (7/14) of participants removed catheters at the 12th week, and none of them experienced re-catheterization in the 24-week follow-up. No serious adverse event was reported. Time-to-event analysis estimated that over 50% patients could respond to EA of more than 8 weeks. Subgroup analysis showed that participants with baseline PVR ≥300 ml and CUR duration ≥12 months experienced longer EA duration to reach the response rate of 50%, compared with those whose PVR <300 ml and CUR duration <12 months (median value: 12 weeks vs. 8 weeks, 12 weeks vs. 4 weeks, respectively).

**Conclusions:**

EA could reduce PVR for pediatric patients suffering from CUR following lumbosacral surgeries, with long-term efficacy and safety. EA treatment of more than 8 weeks was reasonable. Further study of a larger sample and controlling is needed.

**Clinical Trial Registration:**

www.chictr.org.cn, identifier, ChiCTR1800020222.

## Introduction

Chronic urinary retention (CUR) caused by lower motor neuron lesions (LMNL) is a common but lingering condition among pediatric patients after the surgeries of lumbosacral diseases (1, 2). For example, sacrococcygeal teratoma (SCT), the most common extragonadal solid neonatal tumor, can cause lower urinary tract dysfunction (LUTD) either by direct mass effect, or by sequelae related to surgical resection (3). As reported by previous studies, approximately 20%–50% pediatric patients with SCTs suffered from LUTD, of which CUR accounted for 30%, occurring pre-operatively and post-operatively (4–6).

Clinically, CUR means chronically incomplete bladder emptying and large post void residuals (PVR), leading to recurrent urinary tract infections (UTI), stone formation, hydronephrosis and eventual renal deterioration in the long term (1). LUTD involving CUR can result in low self-esteem, social isolation, impaired interpersonal interactions, and behavioral changes including learning difficulties in children (7–9). Besides, parents’ depression symptoms and poor quality of life significantly correlated with behavioral problems in their children with LUTD (10). Therefore, the therapy options for pediatric CUR worth careful attention.

First-line therapy for pediatric CUR includes clean intermittent catheterization (CIC), which is performed several times daily to empty the bladder (11, 12). However, recurrent UTI and urethral stricture formation are chief risks of CIC (13). Besides, lifelong catheterization imposes a major burden on children and their caregivers (14, 15). Sacral neuromodulation (SNM) was approved by US Food and Drug Administration for its effect of relieving lower urinary tract symptoms. However, when using SNM, pediatric patients with neurogenic urinary retention could experience many issues of efficacy and safety, including an often-mixed urodynamic picture from the bladder and urethra, as well as technical drawback related to body growth and neurological stability (16, 17). Also, it's a dilemma that pediatric patients after SNM face a large possibility of reoperation, when coming up with technical faults, pain or discomfort complications (18).

Electroacupuncture (EA) refers to the practice of inserting multiple fine needles with the addition of electrical stimulation at acupoints, which have specific effects on symptoms, according to Traditional Chinese Medicine (TCM) theory (19). The efficacy and safety of EA have been testified in adult patients with urinary disorders, such as urinary incontinence, overactive bladder, and urinary retention (20–24). By needling within the skin and muscles that supplied by the nerves from the pelvic, pudendal and hypogastric nerve roots, which are directly related to the lower urinary tract, EA may influence bladder function through signaling changes from nervous system to the bladder (25–27). EA has been applied in pediatric diseases with long-term outcomes, such as autism spectrum disorders and anorexia, but the research about pediatric chronic urinary retention is scarce (28, 29). Without precedent, this pilot case-series study aims to preliminarily evaluate the effect and safety of EA in treating CUR caused by LMNL following lumbosacral surgeries in pediatric patients, with reasonable EA treatment duration explored.

## Methods

### Study design and participants

This was a single-centre and prospective case-series study performed at Guang’anmen Hospital, China Academy of Chinese Medical Sciences from August 5, 2017, to July 31, 2022. This study was conducted in accordance with the principles of the Declaration of Helsinki and was approved by the Ethics Committee of Guang’anmen Hospital. All participants and their parents or guardians had signed the written informed consent prior to participation. Trial Registration was available at www.chictr.org.cn (Identifier: ChiCTR1800020222).

The inclusion criteria were: (1) being younger than 18 years old, (2) being diagnosed with CUR caused by LMNL (e.g., sacral plexus, cauda equina, or sacral cord lesions) following lumbosacral surgeries, (3) being unable to void and dependent on catheterization including CIC or indwelling catheterization (IC), (4) CUR had lasted for 3 months or longer.

The exclusion criteria were: (1) bladder outlet obstruction uneliminated, (2) urinary system tumors or stones not removed, (3) major psychological disorders, (4) previous implantation with a cardiac pacemaker or SNM, (5) EA treatment duration of less than 1 week.

PVR was measured by urethral catheter output after children’ spontaneous urination attempts without catheterization or assisted bladder emptying (including increasing abdominal pressure or other auxiliary manual methods). For children with CIC, PVR was the average value of 2 measurements. The interval between two measurements should range from 6 h to 3 days.

### Interventions

Bilateral Ciliao (BL32), Zhongliao (BL33), Huiyang (BL35), Shenshu (BL23), and Sanyinjiao (SP6) were inserted with needles. The parameter of needles used at BL32, BL33 and BL35 were 0.30 mm in diameter and 75 mm in length; the needles at BL23 and SP6 were 0.30 mm in diameter and 40 mm in length (Hwato Brand, Suzhou Medical Appliance Factory, China). Bilateral BL32 and BL33 were needled to a depth of 50 mm–70 mm with an angle of 60°–75° inward and downward, into the second and third sacral foramen. Bilateral BL35 were needled to a depth of 50 mm–60 mm with a slightly superolateral direction. Bilateral BL23 and SP6 were needled vertically to a depth of 25 mm–30 mm. The electric stimulators (SDZ-V electroacupuncture apparatus, Suzhou Medical Appliance Factory, China) were transversely connected to bilateral BL32, BL33, and BL35 with a continuous 5-Hz wave (5–10 mA intensity), while connected to bilateral SP6 with a 10 Hz wave (1–2 mA intensity), with an adjustable current intensity to adapt participants’ tolerance. Needles were retained for 30 min during each treatment session. The participants were treated with EA three sessions a week for consecutive 2–16 weeks. In general, PVR less than 5 ml in children was considered normal and not associated with UTI (30). So, when the participant's PVR had reduced to 5 ml within 16 weeks, EA treatment could be terminated depending on the decisions of their parents or guardians and urologists. If the participant's PVR did not reduce to 5 ml, EA treatment was proceeded till the 16th week. Meanwhile, CIC or IC was administered for all participants.

### Outcomes

Responders were defined as participants achieving a reduction of 50% or more in PVR from baseline (31, 32). The primary outcome was the response rate at the 12th week. The secondary outcomes were the change in PVR, in the proportion of participants with severe difficulty in urination and in the proportion of participants with stool retention from baseline, as well as the proportion of participants with catheter removal and the Patient Global Impression of Improvement (PGI-I) score of 1 or 2 at the 12th week. In addition, the proportion of participants without catheterization, the incidence of recurrent symptomatic UTI, hydroureter, and hydronephrosis were assessed in the 24-week follow-up.

Difficulty in urination was divided into four degrees: none, mild, moderate, and severe, which was a subjective grading system for patients suffering from urinary retention as used in previous publications (22). The PGI-I assessment has potential scores of 1–7 representing much better, moderately better, a little better, no change, a little worse, moderately worse and much worse respectively, with a lower score corresponding to more symptom improvement (33). Symptomatic UTI was diagnosed by positive urine culture in a child with isolated fever or urinary symptoms suggestive of UTI. Positive urine culture was defined by the presence of >10^5^ colony forming units of a single organism per milliliter from a clean catch urine sample (34). Recurrent symptomatic UTI was defined as two or more episodes of symptomatic UTI within 24 weeks (35). The Rome IV criteria for children of a developmental age of at least 4 years were used to diagnose stool retention; stool retention was considered present when the child presented at least two of the six symptoms for a minimum of 1 month (36). Hydroureter and Hydronephrosis were scanned by abdominal ultrasound.

As different participants were likely to respond to different EA treatment duration, the response rate was also regarded as a time-to-event outcome in our study. Time-to-event analysis was used to explore the association between the response rate and EA treatment duration.

### Subgroup analysis

Considering that the recovery of bladder voiding function could be affected by the factors of baseline PVR and CUR duration of patients, subgroup analysis was performed to explore the association between the factors and the primary outcome or time-to-event outcome, and to figure out the potential factors affecting the efficiency of EA in treating CUR caused by LMNL following lumbosacral surgeries among pediatric patients (14, 37, 38).

### Safety assessment

All adverse events (AEs) were appropriately managed, monitored, and documented by investigators, and were categorized by the acupuncturist and urologists as either acupuncture related or not acupuncture related within 24 h of occurrence. Serious AEs were immediately reported to the principal investigator (L.Z.S.) and the institutional review board at the clinical sites within 24 h.

### Statistical analysis

Skewed quantitative variables are expressed as median (IQR), and normally distributed variables as mean (SD). The distributions of quantitative variables were assessed for normality by use of the skewness and kurtosis test, and *P* ≥ 0.05 was thought normally distributed. Paired *t*-test or paired Fisher exact test were used to compare pre- and post-treatment data for quantitative and binary variables, respectively. Comparison of binary outcomes between subgroups was performed by independent-samples *t*-test or Fisher exact test. Time-to-event outcome was summarized by Kaplan–Meier estimates. Comparison of time-to-event outcome between subgroups was performed by Log-rank test. Two-sided *P* value less than 5% was considered significant. Statistical analyses were conducted using STATA software version 14.0 (Stata Corp, TX) for Windows.

## Results

### Baseline characteristics

From August 5, 2017 to July 31, 2022, a total of 21 patients were screened, of whom, 7 patients were excluded because of brain tumor resection surgeries (*n* = 1), neuritis (*n* = 2), myelitis (*n* = 2), treatment duration less than 1 week (*n* = 2) (Supplementary Figure S1). A total of 14 participants (mean [SD] age, 12 [4] years; 4 boys [29%]; 10 girls [71%]) were included in the analysis. The most common etiology was the tethered cord syndrome surgery in 6 patients (43%), followed by the lumbosacral vertebra fracture surgery in 5 patients (36%). Lesion location focused on cauda equina [12 participants (86%)] and sacral cord [2 participants (14%)]. Baseline characteristics of 14 participants were shown in Table 1.

**Table 1:** Baseline characteristics.

Characteristics	Participants (*n* = 14)
Age, mean (SD), year	12.5 (4.2)
Boys, *n* (%)	4 (28.6)
PVR, mean (SD), ml	342.9 (319.3)
PVR, *n* (%)	
≥300 ml	5 (35.7)
<300 ml	9 (64.3)
Duration of CUR, mean (SD), m	54.9 (53.5)
Duration of CUR, *n* (%)	
≥12months	10 (71.4)
<12 months	4 (28.6)
Etiology, *n* (%)	
Tethered cord syndrome surgery	6 (42.9)
Lumbosacral vertebra fracture surgery	5 (35.7)
Lumbar teratoma surgery	1 (7.1)
Sacral canal cyst surgery	1 (7.1)
Spina bifida surgery	1 (7.1)
Lesion location, *n* (%)	
Cauda equina	12 (85.7)
Sacral cord	2 (14.3)
Catheterization type, *n* (%)	
CIC	11 (78.6)
IC	3 (21.4)
Difficulty in urination, *n* (%)	
None	0 (0)
Mild	0 (0)
Moderate	1 (7.1)
Severe	13 (92.9)
Stool retention, *n* (%)	5 (35.7)

PVR, post void residuals; CUR, chronic urinary retention; CIC, clean intermittent catheterization; IC, indwelling catheterization.

### Treatment outcomes

**Figure 1:**
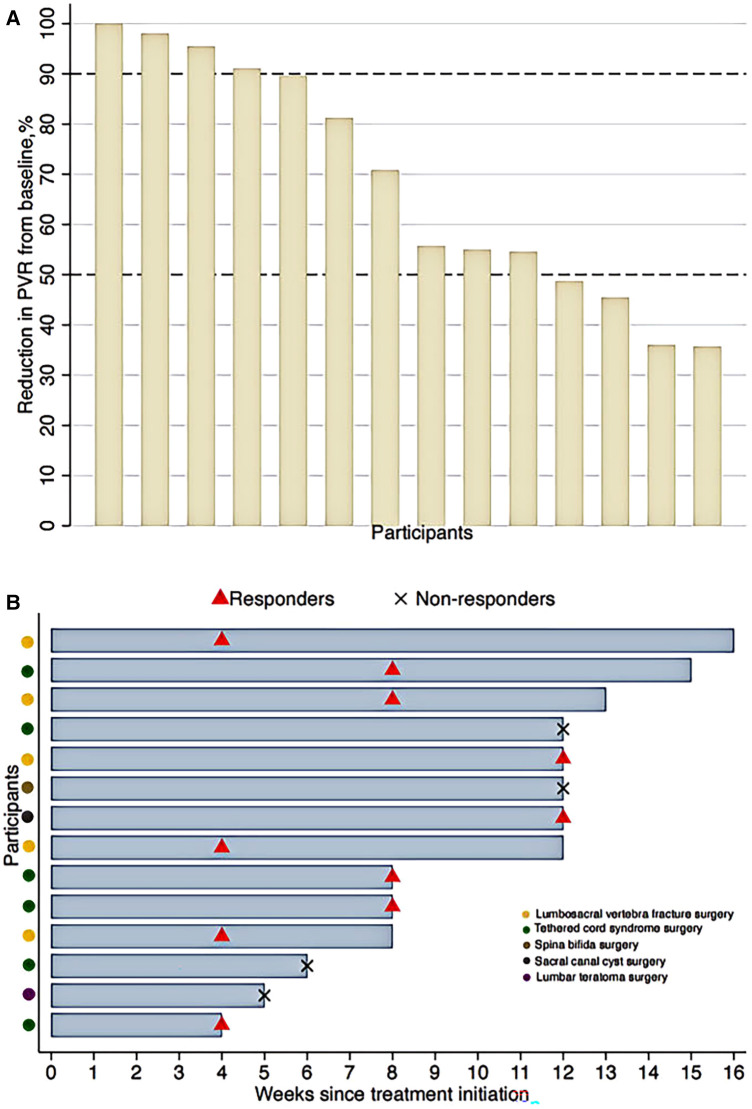
(**A**) Reduction in PVR from baseline after electroacupuncture treatment at the 12th week. (**B**) Electroacupuncture treatment duration. PVR, post void residuals. Responders were defined as participants achieving a reduction of 50% or more in PVR from baseline.

**Figure 2:**
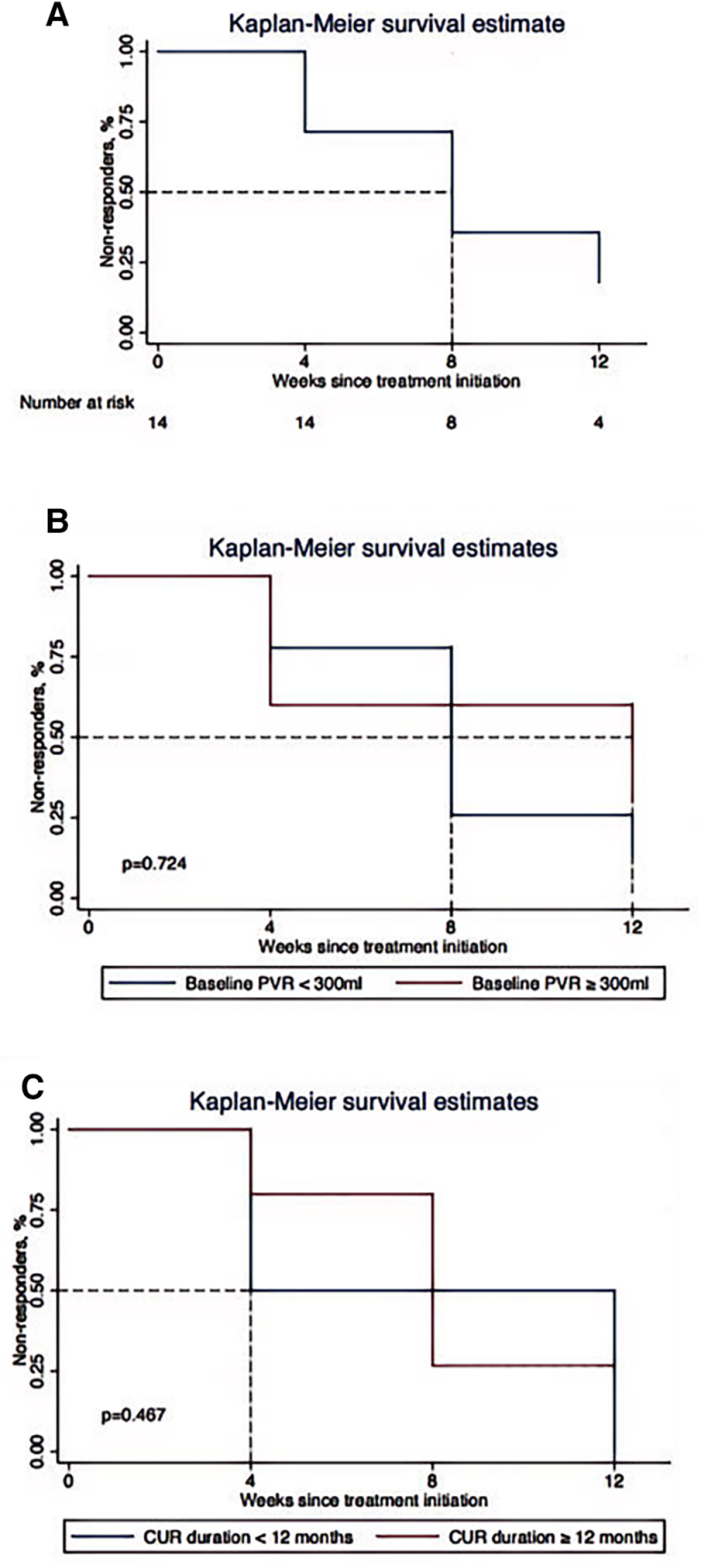
Kaplan–Meier survival estimate for the association between electroacupuncture treatment duration and the proportion of non-responders. (**A**) Overall patients. (**B**) Participants with baseline PVR ≥300 ml and PVR <300 ml. (**C**) Participants with CUR duration ≥12 months and CUR duration <12 months. PVR, post void residuals; CUR, chronic urinary retention. Responders were defined as participants achieving a reduction of 50% or more in PVR from baseline.

After EA treatment, the response rate was 71% (10/14) at the 12th week (Table 2). Around 29% of participants (4/14) achieved a reduction of 90%–100% in PVR from baseline (Figure 1A). As shown in Figure 1B, 4 participants (29%) had responded to EA treatment within 4 weeks. Kaplan–Meier survival curve estimated that 50% participants responded to 8-week EA treatment and depicted a negative trend between the proportion of non-responders and EA treatment duration (Figure 2A).

**Table 2:** Treatment outcomes at the 12th week.

	Outcomes (*n* = 14)	Change from baseline	*P* value
PVR (95% CI), ml	96.8 (30.6, 163.0)	−246.1 (−411.7, −80.5)	0.007
Severe difficulty in urination, *n* (%)	3 (21.4)	−71.4 (−102.2, −40.6)	0.002
Stool retention, *n* (%)	3 (21.4)	−14.3 (−48.4, 19.8)	0.625
Responders, *n* (%)	10 (71.4)	—	—
PGI-I score of 1 or 2, *n* (%)	9 (64.3)	—	—
Catheterization, *n* (%)	7 (50.0)	—	
Recurrent UTI, *n* (%)	0 (0)	—	—
Newly emerging hydroureter or hydronephrosis, *n* (%)	0 (0)	—	—

Responders were defined as patients achieving a reduction of 50% or more in PVR from baseline.

PVR, post void residuals; UTI, urinary tract infections; PGI-I, the patient global impression of improvement.

As displayed in Table 2, at the 12th week, PVR significantly reduced by 246 (95% CI: 80, 412) ml, with an average of 68% reduction from baseline (*P* < 0.001). Likewise, the proportion of participants with severe difficulty in urination was 21% (3/14) with an average of 71% reduction from baseline (*P* < 0.001). Regarding the proportion of participants with stool retention, it just reduced by 14% from baseline (*P* = 0.625). After EA treatment, PGI-I score of 1 or 2 corresponding to much better or moderately better was seen in 9 participants (64%). The proportion of participants with catheter removal was 50% (7/14) at the 12th weeks, and none of these participants experienced re-catheterization in the 24-week follow-up. None of the 14 participants had evidence of recurrent UTI, newly emerging hydroureter or hydronephrosis.

No significant differences were detected in subgroups of baseline PVR and CUR duration in terms of the primary outcome (Supplementary Table S1). However, regarding time-to-event outcome, as illustrated by Figures 2B,C, participants with baseline PVR ≥300 ml and CUR duration ≥12 months experienced a longer EA treatment duration to attain the response rate of 50%, compared with participants whose PVR <300 ml and CUR duration <12 months (median value: 12 weeks vs. 8 weeks, 12 weeks vs. 4 weeks, respectively). It should be noted that although median EA treatment duration was differentiated between subgroups, *P* value was not significant attested by Log-rank test.

### Safety

No episode of serious AEs was reported. Acupuncture-related AEs were infrequent, mild, and transient, which occurred in 2 participants (14%), including sharp pain in 1 participant and localized pigmentation in 1 participant of all cases. No participants discontinued EA treatment because of AEs.

## Discussion

To our best knowledge, this is the first time to evaluate the efficacy and safety of EA in treating CUR caused by LMNL following lumbosacral surgeries among pediatric patients and explore the reasonable treatment duration. We found that EA may be a potential treatment option in reducing PVR and facilitating the recovery of bladder voiding function, with long-term efficacy and safety. The reasonable EA treatment duration should be more than 8 consecutive weeks.

Typically, the bladder processes the urine in two phases: the filling phase followed by the micturition phase. In a child with voluntary voiding, the micturition phase is initiated through complex neural pathways involving the sacral cord micturition centers at the conus medullaris, cortical and pontine centers in the brain, with final signaling passing through the sacral cord micturition centers (S2–S4) (39). This signaling cascade leads to the relaxation of the urethral and internal sphincters and contraction of the detrusor muscle, leading to full bladder voiding (40). It was usual to see CUR caused by LMNL following lumbosacral surgeries, where sacral cord micturition centers and the other lower motor neurons related to micturition are affected. Injuries to cauda equina or sacral spinal cord could result in detrusor underactivity, acontractile detrusor, or detrusor areflexia, which make it impossible for patients to empty the bladder spontaneously and adequately, giving rise to large PVR (41). It is generally accepted that large PVR is the primary problem to be solved in the management of CUR (42). Life-long catheterization to empty the bladder imposes a major burden on children and their caregivers (14, 15). The published experience on the use of SNM in children with neurogenic UR is limited. Sharifiaghdas (43) presented a single-center experience from a small series of pediatric patients with neurogenic LUTD related to lumbosacral congenital anomalies and incomplete spinal cord injury, reporting that positive clinical response (>50% improvement in symptoms) was achieved in seven patients (85%), but urinary retention and the other LUTD were not differentiated significantly in this study.

Therefore, the results of the history research are not comparable with this study considering the heterogeneity of different LUTD syndromes and neurologic lesions.

It is inspiring that this study shows that EA was effective in reducing PVR by 50% or more in 71.4% of pediatric patients with CUR caused by LNML following lumbosacral surgeries. Moreover, 50% (7/14) participants in our study became capable of stopping catheterization at the 12th weeks and none of these participants experienced re-catheterization in the 24 weeks of follow-up, suggesting EA may be effective in maintaining satisfactory bladder voiding function in the long-term. The mechanism of EA may be based on EA stimulation at BL32 and BL33, activating S2–3 afferent nerve fibers to promote detrusor smooth muscle contractions (22), although the other uncertain mechanisms require further investigation. No serious adverse event occurred after EA treatment. Above all, compared to life-long catheterization or permanent SNM electrodes implantation along with reoperation possibilities among pediatric patients, EA could be an option of treatment which is much safer and more convenient with less economic or socio-familial burden.

In addition, our subgroup analysis showed that the response rate at the 12th week was not significantly different across the subgroups of baseline PVR and CUR duration, indicating EA might have relative efficacy and general applicability regardless of the baseline characteristics of patients. Our time-to-event analysis estimated that over 50% participants could respond to EA treatment after 8 weeks of treatment, which was reasonable and consistent with most previous studies (22, 44). Subgroup analysis showed that participants with baseline PVR ≥300 ml or CUR duration ≥12 months experienced a longer EA treatment duration to reach the response rate of 50%, compared with participants whose PVR <300 ml or CUR duration <12 months (median value: 12 weeks vs. 8 weeks, 12 weeks vs. 4 weeks, respectively). It is generally accepted that the neuronal regeneration and reflexes reorganization won’t be easy if the duration of neurogenic CUR following trauma or surgery exceeds 12 months (16). EA could also facilitate the recovery of bladder voiding function if treatment duration is prolonged, even if CUR duration was more than 12 months. Although median EA treatment duration was differentiated between the two subgroups, *P* value was not significantly attested by Log-rank test, which may be due to small sample size in the single center trial. A multi-center randomized controlled trial of a lager sample should be designed to testify these results. Nevertheless, it did highlight an important knowledge gap regarding the clinical decision of EA treatment and its treatment duration basing on different baseline characteristics of pediatric patients.

### Strengths and limitations

This was the first clinical study to evaluate the efficacy and safety of EA in treating CUR caused by LMNL, following lumbosacral surgeries in pediatric patients. This study drew a conclusion that EA could reduce PVR and facilitate the recovery of bladder voiding function. But it has several limitations. Firstly, urodynamic examination was not conducted although PVR was regarded as the primary objective indicator of the outcome. Secondly, control group was not set as this study was to observe the effectiveness of EA in advance. Sham EA control should be set in further study to eliminate the possible interferences of placebo effects and natural course of disease. Thirdly, this was a single-center case series study of a small sample size, and a multi-center randomized controlled trial of a lager sample size should be designed in the future.

## Conclusions

EA could be a potential treatment option for pediatric patients diagnosed with CUR caused by LMNL following lumbosacral surgeries. EA may be effective in reducing PVR and facilitating the recovery of bladder voiding function, with long-term efficacy and safety. The reasonable treatment duration of EA should be more than 8 consecutive weeks. Further study of a larger sample and controlling group is needed.

## Data Availability

The original contributions presented in the study are included in the article/**Supplementary Material**, further inquiries can be directed to the corresponding author.
