# Supplementary material for: Effects of electroacupuncture on pediatric chronic urinary retention: a case-series study
Source: Front Pediatr. 2023 Jul 21;11:1194651. doi: 10.3389/fped.2023.1194651 (PMC10401264; doi:10.3389/fped.2023.1194651)
Supplement: Supplementary file 1 [file Table1.docx]

Supplement Figure 1. Study overview.

treatment duration < 1 weeks (n=2)

14 included in the analysis

16 enrolled

brain tumor resection surgeries (n=1) neuritis (n=2)

myelitis (n=2)

21 patients screened for eligibility

Supplement Table1. Subgroup analysis of the primary outcome.

|  | Responders (n=10) | Non-responders (n=4) | P value |
| --- | --- | --- | --- |
| Baseline PVR, n (%) |  |  | 0.481 |
| ≥300 ml | 3 (30) | 2 (50) |  |
| <300 ml | 7 (70) | 2 (50) |  |
| CUR duration, n (%) |  |  | 0.135 |
| ≥12 months | 6 (60) | 0 (0) |  |
| <12 months | 4 (40) | 4 (100) |  |

Abbreviations: PVR, post void residuals; CUR, chronic urinary retention.
